# Keep palm and carry on: Limited role of thermal acclimation in the invasion of a palm species in Central Europe

**DOI:** 10.1093/plphys/kiaf603

**Published:** 2025-11-20

**Authors:** Alice Gauthey

**Affiliations:** Assistant Features Editor, Plant Physiology, American Society of Plant Biologists; Birmingham Institute of Forest Research, University of Birmingham, Edgbaston B15 2TT, UK

You have probably heard the name “water hyacinth” or “kudzu”; maybe you have even spotted them taking over a pond or creeping across a field near you. These fast-growing invasive plant species seem to be proliferating due to their capacity to thrive in a warmer climate, slowly replacing native species. But is that always how the story goes? Is a plant's success as an invader necessarily tied to its ability to grow better under rising temperatures?

Research suggests that many invasive plants have a competitive advantage that may not only be based on their capacity to thrive at high air temperatures (T_air_) but rather because of their capacity to acclimate their physiological traits to a broad range of T_air_. This thermal acclimation is mainly driven by the acclimation of photosynthesis and respiration (Yamori et al. 2014), which enhance carbon uptake while reducing carbon loss, ultimately leading to greater productivity and competitiveness. Through larger thermal acclimation, the breadth of thermal tolerance could be enhanced and give invasive species a distinct advantage under climate warming (Godoy et al. 2011; Hou et al. 2014), enabling them to expand their distribution.

Conversely, native plant species may also adjust to rising T_air_ by shifting either their maximum rate of photosynthesis (photosynthetic optimum, A_opt_) or the temperature at which A_opt_ occurs (temperature optimum, T_opt_) to higher values. Usually, respiration increases exponentially with higher T_air_ due to elevated metabolic activity, leading to drastic carbon loss. However, acclimation to warmer conditions could involve a reduction in the respiration rate at 25 °C (R25) or temperature sensitivity of respiration (Q10), thereby limiting carbon loss (Atkin and Tjoelker 2003; Crous et al. 2022) and offering a potential mechanism to compete with warm-adapted invasive species. The extent of this acclimation, however, is also influenced by resource availability, such as nutrients and light, which invasive species often utilize more efficiently (Mcalpine et al. 2008; Matzek 2011). Eventually, the outcome of this physiological adjustment will determine whether native or invasive species prevail in what could be described as a “Red Queen's race” for thermal acclimation.

In Central Europe, the evergreen palm *Trachycarpus fortunei*, native to southeastern China, has become a remarkably successful invasive species (Conedera et al. 2018). Initially introduced as an ornamental plant, it now thrives in the sub-Mediterranean climate of the southern Alps. This palm is thought to outcompete native species such as *Ilex aquifolium* (evergreen) and *Tilia cordata* (deciduous) due to its capacity to thrive in warmer conditions. However, whether this thermal advantage arises from physiological acclimation, specifically, a higher optimum temperature for photosynthesis (T_opt_, A_opt_) or lower respiration (R25, Q10) under elevated T_air_, remains unclear.

In a recent publication in *Plant Physiology*, Juillard et al. (2025) explored the mechanisms underlying the invasiveness of *T. fortunei* in Central Europe. The authors set up a transplant experiment in 5 European sites encompassing a wide range of T_air_ and vapor pressure deficit (VPD), where they grew *T. fortunei*, *I. aquifolium*, and *T. cordata* seedlings. Over the course of a year, they measured key physiological and respiratory parameters (T_opt_, A_opt_, R25, Q10) and modeled the whole-plant carbon budget for each species. As expected, A_opt_ and T_opt_ increased at warmer sites for all species (Fig. A). The authors observed that T_opt_ rose by approximately +0.3 °C to +0.8 °C per 1 °C of T_air_, values consistent with previously reported values for thermal acclimation (Choury et al. 2022). Interestingly, T_opt_ increased by +0.6 °C per °C of T_air_ in the two evergreen species, while the deciduous *T. cordata* showed a much weaker response with an increase of only +0.3 °C per °C. This pattern suggests that species with short-lived tissues (e.g. deciduous species) have limited their thermal acclimation potential. In contrast, evergreen species whose foliage can live multiple years and thus experience a wider range of T_air_ may benefit from a higher T_opt_, which would enable photosynthesis across a broader range of temperature conditions. Similarly to T_opt_, A_opt_ also increased at warmer sites. However, since A_opt_ was measured under low VPD conditions (where stomata are not fully open), the authors cautioned that the higher VPD usually associated with higher T_air_ could significantly reduce A_opt_, masking its true acclimation potential. That said, the invasive species *T. fortunei* did not exhibit a larger shift with T_air_ either in A_opt_ or T_opt_ compared with native species.

**Figure.**
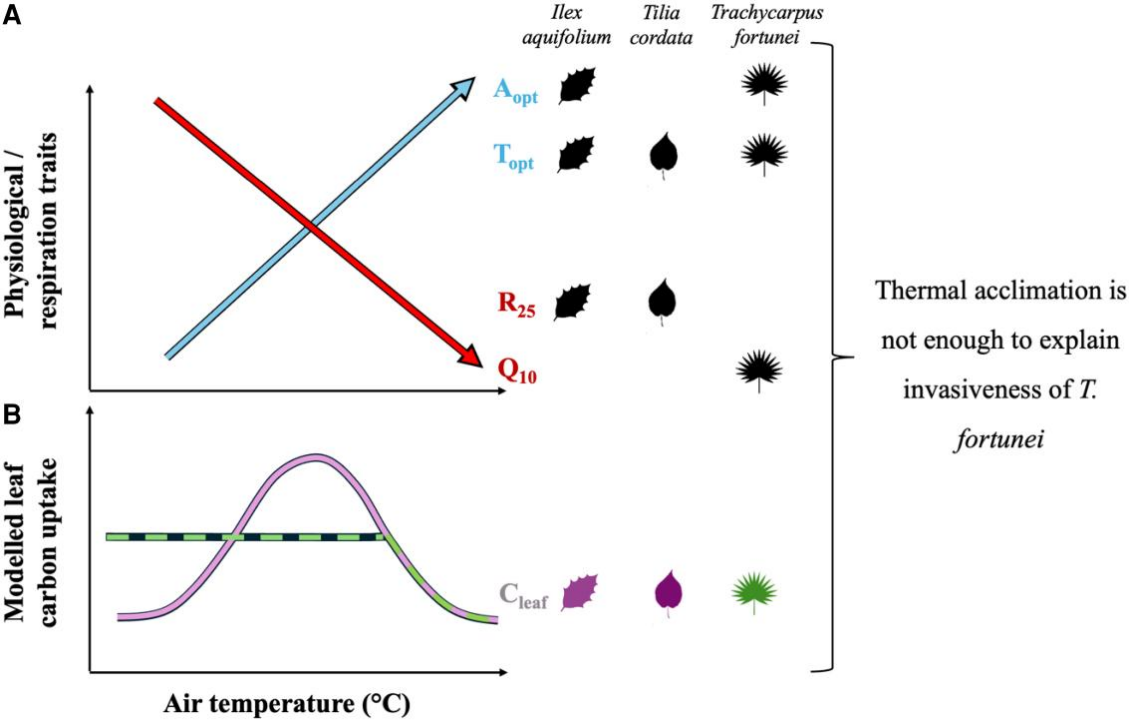
**A)** Simplified relationship between physiological and respiratory traits and air temperature (°C) for A_opt_, T_opt_, R25, and Q10. Positive (blue) and negative (red) relationships are indicated by arrows. **B)** Simplified relationship between modeled leaf carbon (Cleaf) uptake and air temperature (°C). Colors associates species-specific type of response. In both (A) and (B), leaf silhouettes for each species represent the presence or absence of the response.

As previously discussed, thermal acclimation does not always translate into a higher carbon gain; it can also signify a reduced carbon loss via lower respiration at higher T_air_. In *T. fortunei*, while R25 did not adjust to the warmer local T_air_, Q10 decreased at warmer sites (Fig. A). The opposite trend was exhibited by the native species whereby R25 was significantly reduced with T_air_ but no change in Q10 was observed. The authors hypothesized that the invasive species' low but stable R25 could reflect a stable maximum photosynthetic carboxylation rate or the absence of acclimation pressure when T_air_ does not surpass 30 °C. Conversely, Q10 acclimation could reduce carbon loss at warmer temperatures (Atkin et al. 2005; Crous et al. 2022) and increase the thermal breadth of *T. fortunei*, possibly sustaining photosynthetic activity at a wider range of T_air_ than its co-occurring species. Indeed, it was previously shown that invasiveness was positively associated with an expansion of the geographic niche, indicating that invasive species may be able to thrive across a broader range of environmental conditions than co-occurring natives (Higgins and Richardson 2014).

Overall, while warmer climate led to an increase in A_opt_ and T_opt_ and, depending on species, a reduction in R25 and Q10, the only difference found for the invasive species was a lower Q10 at higher T_air_. Juillard et al. therefore concluded that this limited photosynthetic and respiratory adjustment to temperature did not play a central role in the invasiveness of *T. fortunei*.

To further assess the temperature effect on the annual leaf-level carbon (Cleaf) uptake, Juillard et al. used a soil-plant-atmosphere continuum model that estimated Cleaf based on environmental and physiological parameters. The authors incorporated an acclimation potential into the model to test whether local adjustments of photosynthesis or respiratory processes were able to enhance Cleaf. Surprisingly, in *T. fortunei*, Cleaf remained relatively constant across sites and T_air_, showing a significant decline only at the warmer semi-arid site (Fig. B). This contrasted with the other species for which Cleaf increased with higher T_air_, except at the warmest site where Cleaf significantly decreased as well. However, adding the acclimation component to the model resulted in higher Cleaf in *T. fortunei* and *T. cordata*, particularly at milder and warmer sites (respectively), suggesting that photosynthetic and respiratory acclimation may confer benefits to certain species under future climate conditions.

In conclusion, the authors found that although species were able to acclimate to higher local T_air_, this acclimation offered limited benefits at the warmest site. This suggests that more thermophilic plant species may eventually replace both native and current invasive species in this region if T_air_ continues to rise. Moreover, the hypothesis that the introduced *T. fortunei* succeeded in invading the southern Alps due to its temperature tolerance was not fully supported. Instead, the authors proposed that other factors such as the competitive resource use or the efficiency of propagule-based reproduction observed in *T. fortunei* may play an essential role in determining this species' invasiveness.

## Data Availability

Data can be found in the highlighted article and its online supplementary material.
